# The return on investment of TB contact tracing in New York City

**DOI:** 10.5588/pha.25.0054

**Published:** 2026-03-06

**Authors:** J. Goldwater, Y. Harris, K. Neustrom, L. Trieu, C. Chuck, L. Gao

**Affiliations:** 1 Laurel Health Advisors, Washington, DC, USA;; 2 New York City Department of Health and Mental Hygiene, New York, NY, USA.

**Keywords:** tuberculosis, cost-effectiveness, TBI, public health infrastructure

## Abstract

**SETTING:**

In 2013, the New York City Health Department analysed its TB contact tracing programme. Despite long-term declines, TB remained a persistent public health issue in New York City, necessitating continued investment in prevention strategies.

**OBJECTIVES:**

The aim was to evaluate the financial and public health impact of the TB contact tracing programme by conducting a return-on-investment (ROI) analysis.

**DESIGN:**

The study measured programme costs – including personnel, diagnostics, and follow-up care – against projected savings from averted TB cases. A sensitivity analysis was conducted to assess the impact of varying progression rates from TB infection (TBI) to active TB.

**RESULTS:**

The programme identified 3,250 contacts and prevented 64 potential TB cases through early detection and TBI treatment. This resulted in a 95.13% ROI, meaning that for every dollar invested, nearly another dollar was saved. The ROI increased under assumptions of higher TBI progression rates, reinforcing the programme’s cost-effectiveness.

**CONCLUSION:**

Contact tracing plays a critical role in TB control, especially in urban areas with higher incidence. The evaluation supports sustained investment in public health infrastructure and demonstrates that the model can be applied to other infectious disease programmes for targeted prevention and early intervention.

Globally, TB is the leading cause of death from infectious diseases and there are over 8,000 active TB cases diagnosed annually in the United States (US).^1^ New York City (NYC) accounts for the highest number of TB cases among major cities, comprising 6–7% of all US cases each year.^2^ Following the TB epidemic in the late 1980–1990s, federal, state, and local efforts drastically reduced TB incidence in both the nation and NYC.^3^ These included improved diagnostics, treatment adherence programmes, enhanced infection control, and targeted treatment for high-risk groups, such as those with HIV.^4^ Consequently, NYC saw TB cases drop from 3,755 in 1992 to 534 in 2022.^1^ However, the decline has slowed recently, with a 28% increase in cases in 2023 compared to the previous year, as the city emerged from the SARS-CoV-2 pandemic amidst evolving public health challenges and changing epidemiological trends.^4^ TB infection (TBI) from recent exposure to active TB is a significant risk for developing TB disease.^4^ An estimated 10% of those with TBI progress to active TB within their lifetime, with half developing it within 2 years of exposure.^1^ Contact investigation aims to interrupt TB transmission by diagnosing and treating latent TB infection (LTBI) and identifying and treating active TB early to reduce infectivity.^4,5^

A comprehensive TB control programme can be costly.^6^ While the effectiveness of contact investigation in preventing the spread of TB has been well-documented, fewer studies have examined its cost-effectiveness. A detailed financial analysis comparing the costs associated with TB contact investigations and LTBI treatment against the savings from avoided TB disease treatment revealed that, in most scenarios, the financial investment in contact tracing yields positive returns.^7,8^ Specifically, the analysis highlighted that for every dollar spent on TB contact investigations and LTBI treatment, there was a substantial reduction in future TB disease treatment cost.^9^ The NYC Health Department conducted a comprehensive study to calculate the return on investment (ROI) for its TB contact investigation programme. This enables evidence-based decisions to allocate resources effectively to combat TB transmission and manage health care costs. Given the significant financial burden associated with treating active TB and the potential for transmission in densely populated urban areas, the department sought to quantify the cost-effectiveness of its efforts.

## METHODS

This study calculates the ROI on the NYC Health Department TB contact tracing programme using data from 2013. This year was selected for several reasons. First, TB’s slow progression requires ample time to evaluate public health interventions’ short- and long-term effects. Additionally, staffing levels for the TB control programme were closer to being ‘fully staffed’ in 2013 than in recent years, allowing a more accurate assessment of the programme’s effectiveness and resource use at near-optimal capacity. This understanding is crucial for evaluating the actual costs and benefits of the intervention, as understaffing could skew results. Furthermore, TB case rates in 2013 were similar to current rates, enhancing the relevance of this analysis to present-day TB control efforts. While ethical approval was not required, the analysis relied exclusively on routinely collected programmatic and surveillance data from the NYC Department of Health TB Control Program. All data were de-identified prior to analysis and were used in accordance with applicable data governance, confidentiality, and public health reporting requirements.

The ROI analysis was conducted with several steps to evaluate the costs and potential savings associated with the programme:

1) Quantifying the total costs associated with the TB contact tracing programme.
2) Estimating the number of additional TB cases that would have occurred without the contact tracing programme.
3) Calculating the cost of treating active TB cases.
4) Savings generated by the TB contact tracing programme.

This analysis is intended as a budgetary ROI assessment based on model-derived estimates rather than a causal evaluation of programme effectiveness. Estimates of TB cases ‘prevented’ or ‘averted’ reflect projected outcomes under stated assumptions regarding disease progression, transmission, and treatment uptake in the absence of contact tracing, and should not be interpreted as empirically demonstrated causal effects. The findings, therefore, represent plausible counterfactual scenarios used to inform resource allocation and programme planning rather than direct causal attribution.

### Cost of contact tracing programme

The first step was to quantify the total costs of the TB contact tracing programme, including expenses for implementation and operation: personnel costs, diagnostic testing (tuberculin skin tests and interferon-gamma release assays), administrative costs for data collection and analysis, and other costs like clinical follow-up, medications, travel, and equipment. This study focused on costs related to identifying, evaluating, and preventing TB among household or leisure contacts, given the resource-intensive and high-risk nature of these investigations. Costs associated with exposures in congregate settings and health care facilities were excluded due to significant operational differences.

### Additional TB cases without the contact tracing programme

The second step in the methodology involves estimating the number of additional TB cases that would have occurred without the contact tracing intervention. This rate represents the likelihood that individuals with untreated LTBI will eventually develop active TB over their lifetime. Applying these progression rates to the number of contacts with latent infection allowed for the estimation of how many individuals in the traced population might have developed active TB without intervention. The analysis included first-generation infections, the initial transmission of TB from a primary case to a new host, as well as second-generation infections and cases averted, which occur when a proportion of first-generation infected individuals develop disease and then transmit it to others. According to the Centers for Disease Control and Prevention (CDC), 73% of those individuals who develop active TB disease are potentially infectious.^10,11^ Based on Health Department contact tracing data for 2013, an average of five household or leisure contacts were tested for every TB case identified through contact tracing. Estimation of second-generation TB cases was based on simplified, linear transmission assumptions using established public health parameters, including the proportion of infectious cases and the average number of contacts per case. This approach was selected to provide a transparent and conservative approximation of downstream transmission within the constraints of a budgetary ROI model, rather than to fully characterise complex transmission dynamics. While urban TB transmission is influenced by heterogeneous contact patterns and contextual factors not captured by this framework, the assumptions reflect programme-relevant averages derived from local surveillance data and published estimates. Accordingly, second-generation case estimates should be interpreted as modelled projections intended to approximate potential downstream impacts, not as precise representations of transmission behaviour.

### Cost of treating active TB

After estimating potentially avoided TB cases, the next step is calculating the cost of treating active cases. This analysis covers the financial burden of active TB management, which involves hospitalisation, medications, and directly observed therapy (DOT) where health care workers monitor medication intake. Additionally, public health interventions like intensive case management and ongoing contact tracing are included in the total cost. An average cost per active case is calculated using local and national health care data, reflecting the comprehensive care needed. These costs are then multiplied by the projected number of TB cases without the contact tracing programme, yielding the total cost of treating active TB cases without these efforts.

### Calculating savings by the TB contact tracing programme

The final step is calculating the ROI for the TB contact tracing programme. The cost of averted TB cases is found by subtracting the cost of the contact tracing programme from the total cost of treating the estimated active TB cases that would have arisen without it. This includes all direct medical costs, such as hospitalisation, medications, diagnostic procedures, and DOT. Public health resources would also have been used for contact tracing and controlling the spread of TB if these cases had not been prevented. The expected number of active TB cases averted, multiplied by the average cost of treating one drug-susceptible TB case in NYC, reflects the financial burden on the health care system if the programme had not prevented the progression of TBI to active TB. Patients engaged in medical visits for treatment are not involved in other routine economic activities they would usually participate in. Patient time costs were valued using an average per-capita GDP–based estimate to approximate the opportunity costs of treatment and travel time. This approach does not account for individual employment status, income variability, or differences in time use during hospitalisation and may therefore overestimate opportunity costs for some patients. The valuation was applied as a simplifying, population-level assumption intended to provide an upper-bound estimate of productivity losses rather than a precise measure of individual economic impact. As such, opportunity cost estimates should be interpreted cautiously and considered supplementary to direct medical cost findings. These calculations are included in the analysis under opportunity costs in Table 3. The counterfactual scenario assumes that, in the absence of systematic contact tracing, a substantial proportion of latent and early-stage TB cases would not have been identified or treated in a timely manner. While NYC benefits from a high-capacity health system with routine health care access and passive case detection, passive surveillance alone is less effective at identifying asymptomatic LTBI and early disease among recently exposed contacts. Contact tracing accelerates identification and treatment of these individuals, reducing the likelihood of delayed diagnosis, ongoing transmission, and progression to active disease. Accordingly, the model assumes earlier detection and intervention attributable to contact tracing, rather than a complete absence of diagnosis under passive care pathways.

Data were collected from the NYC Health Department TB programme regarding TB cases, contacts identified, and the costs of the contact tracing programme in 2013; the Centers for Medicare & Medicaid Services 2013 Physician Fee Schedule regarding the costs of treating an active TB patient; and the United States Bureau of Economic Analysis for the 2013 U.S. Gross Domestic Product to estimate loss of patient opportunity costs.

## RESULTS

In 2013, the NYC contact tracing programme identified 3,250 contacts from 650 persons with active, infectious TB. Of these, 807 (25%) had a positive test for TBI; 10 were diagnosed with TB disease at the time of investigation, 5 were subsequently diagnosed with active disease, and 7 contacts without a positive test developed disease at a later point. In 2013, the progression rate of LTBI to active TB was about 10%. At this rate, a predicted 81 individuals would have developed TB in their lifetime without treatment. The 22 contacts who were diagnosed with prevalent TB disease likely would have occurred regardless of contact investigation activities and were excluded from the analysis. This left 59 potential cases, representing the aversion of a first-generation infection. By applying this progression rate, the study estimates how many cases of active TB would have occurred had no public health intervention been undertaken.

### Cost of contact tracing programme

The programme’s total cost, including personnel expenses,^1^ diagnostic testing, follow-up care, and administrative tasks, amounted to $1,695,962 in 2013. The cost breakdown is shown in Table 1.

**TABLE 1.** Detailed costs of NYC Health Department contact tracing programme.

Cost of NYC Health Department staff	$1,061,812
Training cost	$41,185
Computers/equipment/data collection	$82,000
Supply costs	$78,000
Transportation costs	$240,463
Costs of LTBI treatment (270 days)	$192,502
Total	$1,695,962

Source: NYC Health Department Bureau of Tuberculosis Control. TBI treatment includes chest X-rays, TST, sputum spears, cultures, and first-line susceptibility tests at the initial diagnoses and 2-and 3-week intervals. It also includes INH and RFI monotherapy.

NYC = New York City; LTBI = latent TB infection; TBI = TB infection; TST = tuberculin skin test; INH = isoniazid; RFI = rifampin.

### Additional cost of TB cases

The NYC Department of Health and Mental Hygiene contact tracing programme averted 81 TB cases from 807 individuals with positive tests and a 10% progression rate to TB disease. Consequently, 59 first-generation TB cases were prevented after excluding 22 contacts who developed active TB. Untreated, these 59 could lead to second-generation TB cases. According to CDC estimates, 73% of these cases were potentially infectious, preventing 43 infectious cases. With a median of five contacts per case, these 43 could result in 215 additional contacts. Prior data indicate that approximately 25% of contacts (*n* = 54) would be infected, with 10% (*n* = 5) progressing from LTBI to active TB. In total, 64 (59 first-generation and 5 second-generation) TB cases were prevented through the 2013 NYC contact tracing programme. The methodology for calculating averted cases is shown in Table 2.

**TABLE 2.** Averted first- and second-generation TB cases.

	Step	Calculation
1	81 initial cases; 22 cases subsequently diagnosed with TB	81 − 22 = 59 initial cases (potential first-generation cases averted through contract tracing)
2	43 of those initial 59 cases are potentially infectious	59 × 0.73 = 43 cases are potentially infectious (CDC estimate of proportion of infectious cases)
3	Based on NYC Health Department data from 2013, an average of five contacts elicited per infectious case	43 × 5 = 215 contacts (number of contacts identified around potentially infectious cases)
4	Based on NYC Health Department data from 2013, of contacts identified, 25% would be infected	215 × 0.25 = 53.75 (rounded to 54) (number of infected contacts averted)
5	Based on CDC data, there is a 10% progression rate from LTBI to TB within 2 years after the initial infection	54 × 0.10 = 5.4 (rounded to 5) (number of second-generation cases averted)
6	First- and second-generation infection potentially averted through contact tracing	59 + 5 = 64 (sum of steps 1 and 5)

NYC = New York City; CDC = Centers for Disease Control and Prevention; LTBI = latent TB infection.

### Cost of treating active TB cases

The average cost of treating one active drug-susceptible TB case in NYC was $51,709. Table 3 illustrates the costs contributing to this figure.

**TABLE 3.** Overall cost of treating active TB cases (per patient).

Category	Costs
Hospitalisation (10-day stay)	$25,000.00
Initial diagnosis and end of treatment	
Chest X-ray	$30.96
TST	$8.17
Three sputum smears	$16.17
Culture	$6.19
First-line tests	$31.19
Treatment at 2- and 3-week phase	
Three sputum smears	$16.17
Culture	$6.19
Drug dosing	
Initial phase	
Isoniazid	$23.86
Rifampin	$20.48
Pyrazinamide	$144.44
Ethambutol	$27.21
Continuation phase	
Isoniazid	$47.72
Rifampin	$40.96
Patient opportunity costs	
60 min initial diagnosis	$6.00
DOT drug administration	$0.60
30 min transportation	$3.00
Time spent in treatment	$25,900.00
Travel (76 visits/$5.00 a visit)	$380.00
Total cost for TB	$51,709

Source: NYC Health Department Bureau of Tuberculosis Control, Centers for Medicare & Medicaid Services 2013 Physician Fee Schedule, and United States Bureau of Economic Analysis.

TST = tuberculin skin test; DOT = directly observed therapy.

### Savings by the contact tracing programme

The savings from the TB contact tracing programme are the difference between the projected costs of treating active TB without the programme and the actual costs of the programme. Savings from 64 averted TB cases at an average cost of $51,709 per case in NYC totalled $3,309,376, demonstrating the funds the health care system saved due to the programme’s success. These savings are significant due to the high costs of treating active TB and the risk of community spread if untreated. By preventing 70 first- and second-generation active TB cases, the programme lowered immediate treatment costs.

In this analysis, the estimated return on investment was 95.13%, indicating that the TB contact tracing programme in NYC approached cost neutrality while generating meaningful public health benefits. Specifically, for every dollar spent on the programme, approximately $0.95 in TB treatment costs were projected to be offset under base-case model assumptions, resulting in near break-even financial performance rather than a large net fiscal gain. Given uncertainty in several underlying parameters, this estimate should be interpreted as an approximate, scenario-dependent outcome that reflects substantial cost offset rather than precise financial savings. The primary implication is that the programme largely covers its costs through avoided TB treatment expenses, estimated at $1,613,414 for 2013, while delivering significant population-level health benefits.

Sensitivity analysis examined how variation in the LTBI-to-active TB progression rate affects ROI estimates, as shown in Figure. Progression rates ranging from 0.03 to 0.12 produced ROI values from approximately −0.9 at the lowest rate to between 0.2 and 1.5 at rates of 7% and higher, reflecting increasing cost-effectiveness as more cases are intercepted through contact tracing. This parameter was varied because it is both highly uncertain and a primary driver of downstream costs, while other inputs, including contact rates, infectiousness assumptions, treatment timing, and second-generation transmission, were held constant based on empirical data and established estimates. Accordingly, the sensitivity analysis should be interpreted as illustrative rather than exhaustive, and future work could incorporate multi-parameter or probabilistic approaches to more fully characterise uncertainty.

**FIGURE.**
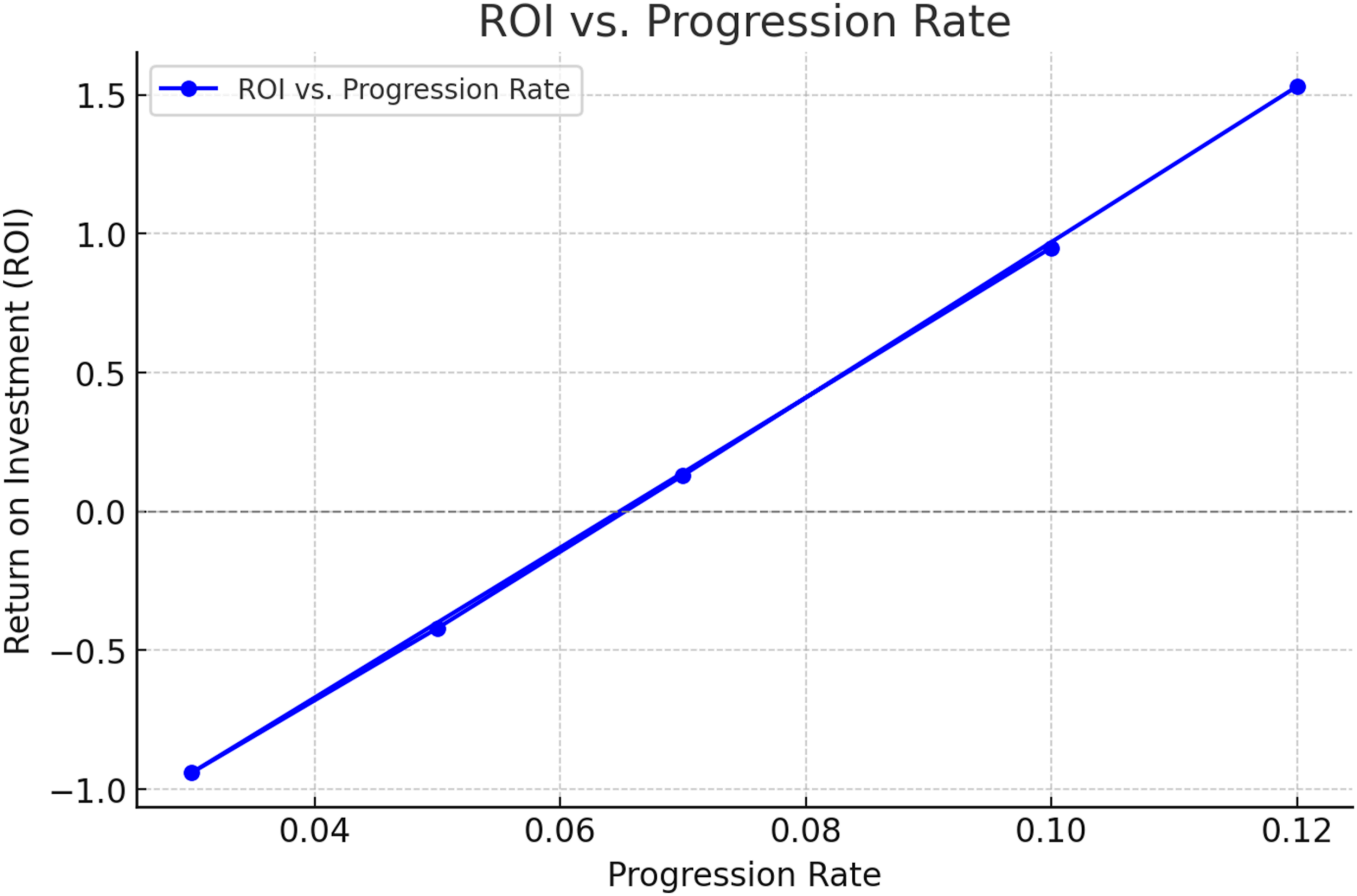
Return on investment vs. progression rate from TB infection to active TB.

## DISCUSSION

The findings of this analysis are most generalisable to large, high-income urban settings with established TB surveillance systems, robust public health infrastructure, and sufficient workforce capacity to conduct comprehensive contact investigations. Jurisdictions with similar population density, health care access, and programmatic cost structures may observe comparable returns on investment from sustained TB contact tracing efforts. However, the magnitude of ROI observed in NYC reflects local epidemiology, labour costs, and health care utilisation patterns that may not be directly replicable elsewhere. In low- and middle-income country settings, differences in TB burden, resource availability, diagnostic access, and surveillance capacity may substantially alter both programme costs and economic returns, underscoring the need for local adaptation of model assumptions.
